# The NF-κB pathway in inflammatory responses in preeclampsia: A systematic review and meta-analysis

**DOI:** 10.1097/MD.0000000000050552

**Published:** 2026-09-18

**Authors:** Hui Zhang, Yanhua Nong, Meiqi Huang, Riyuan Wei, Lihan Feng

**Affiliations:** a Obstetrics Department, The People’s Hospital of Chongzuo, Chongzuo, Guangxi Zhuang, China.

**Keywords:** inflammation, NFκB, preeclampsia, SIRT1, therapeutic targets

## Abstract

**Background:**

Preeclampsia is a pregnancy-specific hypertensive disorder associated with systemic inflammation, endothelial dysfunction, and adverse maternal and fetal outcomes. The nuclear factor kappa B (NF-κB) signaling pathway has been implicated in inflammatory activation, but its role in preeclampsia remains incompletely defined. This systematic review and meta-analysis aimed to evaluate the association between NF-κB pathway activation and inflammatory responses in preeclampsia.

**Methods:**

This meta-analysis reviewed 15 peer-reviewed articles focusing on the involvement of the NF-κB pathway in preeclampsia. Quantitative assessments included changes in systolic and diastolic blood pressure and levels of key inflammatory mediators, including tumor necrosis factor-alpha (TNF-α), interleukin-1 beta, interleukin-6 (IL-6), and NF-κB.

**Results:**

Systolic and diastolic blood pressure were significantly elevated in patients with preeclampsia. TNF-α and NF-κB levels were also significantly increased, indicating enhanced inflammatory activation associated with the disease. In contrast, interleukin-1 beta and IL-6 levels did not differ significantly, although IL-6 showed a nonsignificant trend toward increased levels.

**Conclusion:**

This meta-analysis suggests that NF-κB activation, together with increased TNF-α levels, may contribute to the inflammatory pathophysiology of preeclampsia. These findings support further investigation of NF-κB-related pathways, including Sirtuin 1–mediated regulation, as potential biomarkers and therapeutic targets.

## 1. Introduction

Preeclampsia remains a significant challenge in obstetrics, characterized predominantly by high blood pressure and proteinuria.^[1,2]^ It represents a significant source of morbidity and mortality for both mothers and infants globally, impacting approximately 2%–8% of pregnancies.^[3,4]^ The condition often leads to severe complications such as eclampsia, fetal growth restriction, and even maternal and perinatal death, which indicate its critical impact on public health.^[5–7]^ The condition’s complexity and the severe health implications show the critical need for a deeper understanding and more effective management strategies. The etiology of preeclampsia is multifactorial, including genetic predispositions, immunological factors,^[8,9]^ and environmental triggers.^[10]^ Current theories suggest an imbalance in angiogenic factors, immune maladaptation, and placental ischemia as fundamental contributors to its pathogenesis.^[11,12]^ Despite extensive research, the exact mechanisms triggering these pathological changes remain elusive. Currently, the management of preeclampsia primarily involves symptomatic treatment aimed at controlling blood pressure and preventing seizure activity, with medications such as antihypertensives and magnesium sulfate.^[13]^ However, these treatments do not address the underlying disease mechanisms and only serve to manage the symptoms, rather than prevent the onset of the condition.

At the molecular level, preeclampsia has been associated with profound systemic inflammation,^[14,15]^ endothelial dysfunction,^[16]^ and oxidative stress.^[17]^ In addition, research shows that increased levels of inflammatory markers correlate with the intensity of symptoms and outcomes, suggesting that inflammation may not just be a consequence but could be a driver of the disease.^[18]^ Central molecular factors such as cytokines, including tumor necrosis factor-alpha (TNF-α) and interleukin-6 (IL-6), alongside transcription factors like nuclear factor kappa B (NF-κB), play pivotal roles in mediating the inflammatory responses.^[19]^ Furthermore, the interplay between Sirtuin 1 (SIRT1) and NF-κB offers a distinct approach on controlling inflammatory pathways. SIRT1, known for its regulatory roles in anti-inflammatory processes, modulates the activity of NF-κB.^[20]^ Enhancing SIRT1 activity could offer a therapeutic strategy to mitigate the inflammatory responses observed in preeclampsia. NF-κB, in particular, is crucial in the inflammatory pathways, influencing the expression of various adhesion molecules and pro-inflammatory cytokines.^[21]^ Despite the recognition of inflammation’s role in preeclampsia, detailed explorations into specific inflammatory pathways, such as those mediated by NF-κB, are lacking. Studies have suggested that targeting NF-κB may mitigate inflammatory responses and potentially ameliorate symptoms of preeclampsia, yet comprehensive syntheses of these findings are sparse. This meta-analysis aims to consolidate existing research focusing on the role of NF-κB in preeclampsia. By systematically reviewing the literature and integrating findings from diverse studies, we hope to clarify the contribution of NF-κB to the disease’s pathophysiology. Addressing these aspects could pave the way for the creation of more specialized treatments that not only treat but potentially prevent the onset of preeclampsia, offering significant benefits for maternal and fetal health globally.

## 2. Methods

### 2.1. Search methodology and selection standards

This study was approved by the Ethics Committee of The People’s Hospital of Chongzuo. The systematic review was conducted in accordance with the Cochrane Handbook for Systematic Reviews of Interventions and the reporting guidelines provided by the Preferred Reporting Items for Systematic Reviews and Meta-analyses.^[22]^ A literature review was performed utilizing databases such as PubMed, Embase, the Cochrane Central Register of Controlled Trials, Ovid MEDLINE, and Web of Science. The search was restricted to studies published in English and available until October 20, 2024. The searches were performed using the Medical Subject Headings (MeSH) terms, “Pre-Eclampsia” and “NF-kappa B” and “Sirtuin 1.” The search approach used for PubMed was (((((((((((((((((((((((((((((((((((Pre Eclampsia) OR (Preeclampsia)) OR (Pregnancy Toxemias)) OR (Pregnancy Toxemia)) OR (Toxemia, Pregnancy)) OR (Edema-Proteinuria-Hypertension Gestosis)) OR (Edema-Proteinuria-Hypertension Gestosis)) OR (Gestosis, Edema-Proteinuria-Hypertension)) OR (Hypertension-Edema-Proteinuria Gestosis)) OR (Gestosis, Hypertension-Edema-Proteinuria)) OR (Hypertension-Edema-Proteinuria-Gestosis)) OR (Proteinuria-Edema-Hypertension Gestosis)) OR (Gestosis, Proteinuria-Edema-Hypertension)) OR (Proteinuria-Edema Hypertension Gestosis)) OR (EPH Complex)) OR (EPH Toxemias)) OR (EPH Toxemia)) OR (Toxemia, EPH)) OR (Toxemias, EPH)) OR (EPH Gestosis)) OR (Gestosis, EPH)) OR (Toxemias, Pregnancy)) OR (Toxemia Of Pregnancy)) OR (Of Pregnancies, Toxemia)) OR (Of Pregnancy, Toxemia)) OR (Pregnancies, Toxemia Of)) OR (Pregnancy, Toxemia Of)) OR (Toxemia Of Pregnancies)) OR (Preeclampsia Eclampsia 1)) OR (1, Preeclampsia Eclampsia)) OR (1s, Preeclampsia Eclampsia)) OR (Eclampsia 1, Preeclampsia)) OR (Eclampsia 1s, Preeclampsia)) OR (Preeclampsia Eclampsia 1s)) OR (“Pre-Eclampsia”[Mesh])) AND ((((Silent Mating Type Information Regulation 2 Homolog 1) OR (SIRT1)) OR (“Sirtuin 1”[Mesh])) OR ((“NF-kappa B”[Mesh]) OR (((((((((((((((((((((Immunoglobulin Enhancer-Binding Protein) OR (Enhancer-Binding Protein, Immunoglobulin)) OR (Immunoglobulin Enhancer-Binding Protein)) OR (kappa B Enhancer-Binding Protein)) OR (NF-κB)) OR (Transcription Factor NF-kB)) OR (Factor NF-kB, Transcription)) OR (NF-kB, Transcription Factor)) OR (Transcription Factor NF-kB)) OR (NF-kappaB)) OR (NF-kappaB)) OR (NF-kB)) OR (NF-kB)) OR (Nuclear Factor-Kappab)) OR (Factor-Kappab, Nuclear)) OR (Nuclear Factor-Kappab)) OR (Ig-EBP-1)) OR (Ig-EBP 1)) OR (NF-kappa B Complex)) OR (Complex, NF-kappa B)) OR (NF kappa B Complex)))). The search method was applied to all databases.

In this review, selection criteria were defined to ensure a systematic and rigorous approach in our meta-analysis on the role of NF-κB in preeclampsia. We first screened titles and abstracts to eliminate duplicates and filter out studies that did not focus on NF-κB’s role in preeclampsia. Full-text articles were then assessed for eligibility based on specific inclusion criteria, focusing on studies that quantified NF-κB activity and its correlation with preeclampsia outcomes. We excluded articles that did not provide empirical data, lacked necessary methodological details, or were not in English and review papers. The final selection included studies that met all predefined criteria and were suitable for data extraction and quality assessment using standardized tools.

### 2.2. Data extraction

EndNote 20 was used to organize the studies collected. After removing duplicates, 2 independent researchers meticulously assessed the titles and abstracts of the remaining articles. Only studies that satisfied the inclusion criteria and received consensus approval from both researchers were included in the meta-analysis. Disagreements were collaboratively discussed, and if a consensus was still unattainable, the final decision was reached after consulting with a third reviewer. Data extraction was carried out using a predefined template to maintain uniformity and precision across the dataset. For each research article analyzed, details such as the number of participants, maternal age, gestational age, parity, and body mass index were collected. gender, health status, comparator groups, study duration, systolic and diastolic blood pressure values, and levels of inflammatory markers were collected. Additionally, study characteristics such as the authors’ names, year of publication, and study design were recorded. Quantitative information was taken from the text, tables, and figures and recorded as means ± standard deviation. The GetData Graph Digitizer program was used to accurately extract numerical values from data that were displayed graphically.

### 2.3. Statistical analysis

In order to adequately handle the additional uncertainty resulting from study variability, the pooled analysis employed a random-effects restricted maximum likelihood model to assess the mean differences in each outcome between preeclamptic and healthy pregnant women. Heterogeneity among studies was evaluated using the I^2^ statistic, with values over 50% indicative of significant inconsistency.^[22]^ Publication bias was assessed with a funnel plot, further quantified by Egger’s regression test and Begg’s nonparametric rank correlation test, considering a *P*-value of <.05 as indicative of significant differences. To identify sources of heterogeneity, the Galbraith plot was used. A leave-one-out sensitivity analysis was performed to reevaluate the overall effect when fewer than one-quarter of the studies identified by the Galbraith plot contributed to heterogeneity. All statistical analyses were carried out with Stata version 18.0.

## 3. Results

### 3.1. Literature search and study selection

**Figure 1.**
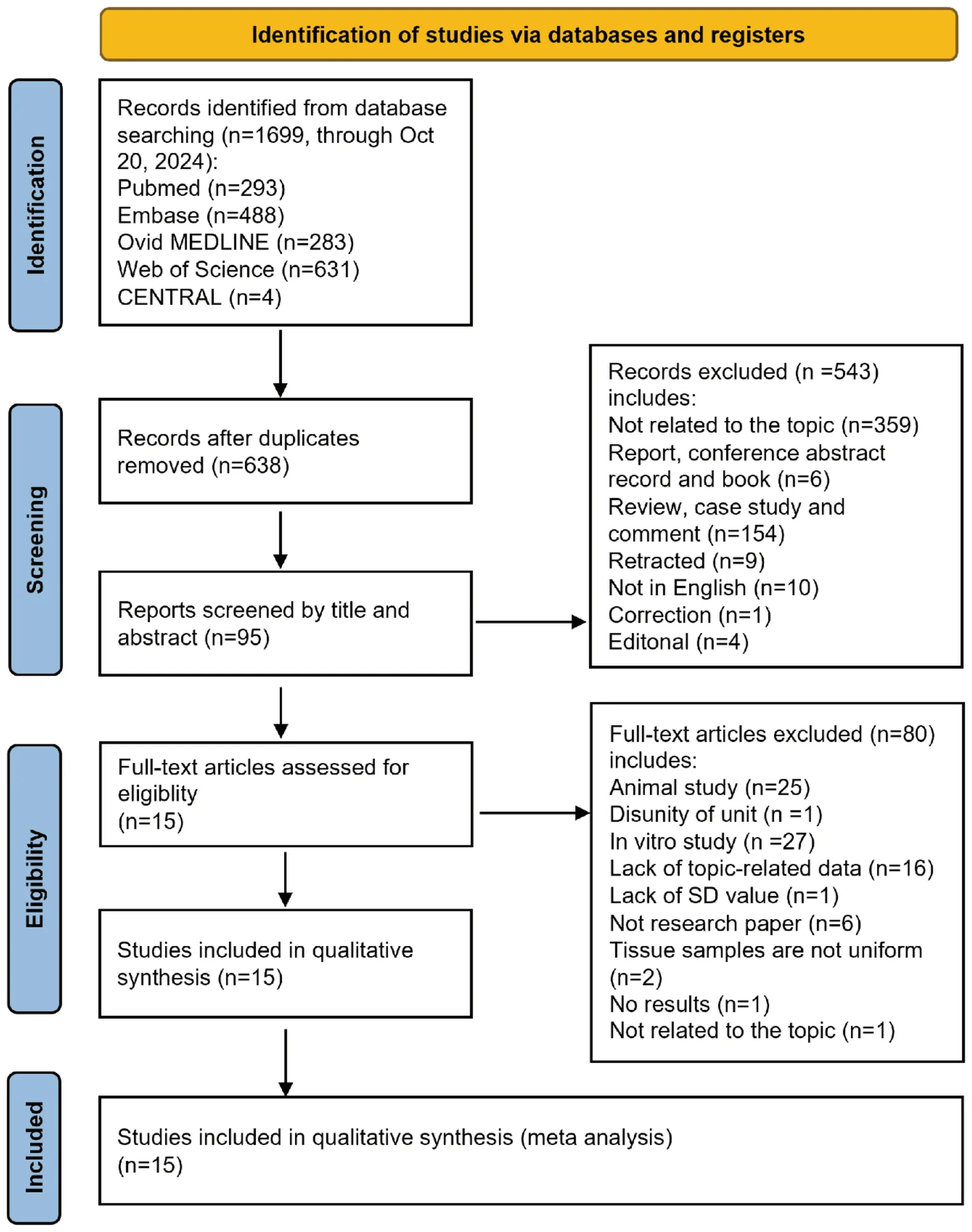
Study selection flowchart following PRISMA guidelines. PRISMA = Preferred Reporting Items for Systematic Reviews and Meta-Analyses.

Initially, our research involved searching 1699 records through databases and references from earlier studies. Following the removal of duplicates, 638 publications were screened by titles and abstracts. Out of these, 543 articles were excluded due to not meeting our specific inclusion and exclusion criteria. Following this, 95 papers underwent full-text review, from which 15 studies fulfilled our criteria and were chosen for detailed analysis (Fig. 1).

### 3.2. Characteristics of included studies

This meta-analysis encompasses 15 studies, involving a total of 3432 participants. The participants predominantly comprised pregnant women diagnosed with preeclampsia, ranging in age from 15 to 43 years. For gestational characteristics, the participants were generally in their second or third trimester, which is critical for studying preeclampsia that typically manifests after the 20th week of pregnancy. Each study provided detailed data on maternal characteristics including body mass index, parity, and specific health conditions linked to preeclampsia. The primary outcomes assessed across the studies included systolic and diastolic blood pressure, levels of specific inflammatory markers like TNF-α, IL-6, and the activation of NF-κB pathways (Table S1, Supplemental Digital Content 1).

### 3.3. The impact of preeclampsia on hypertension

**Figure 2.**
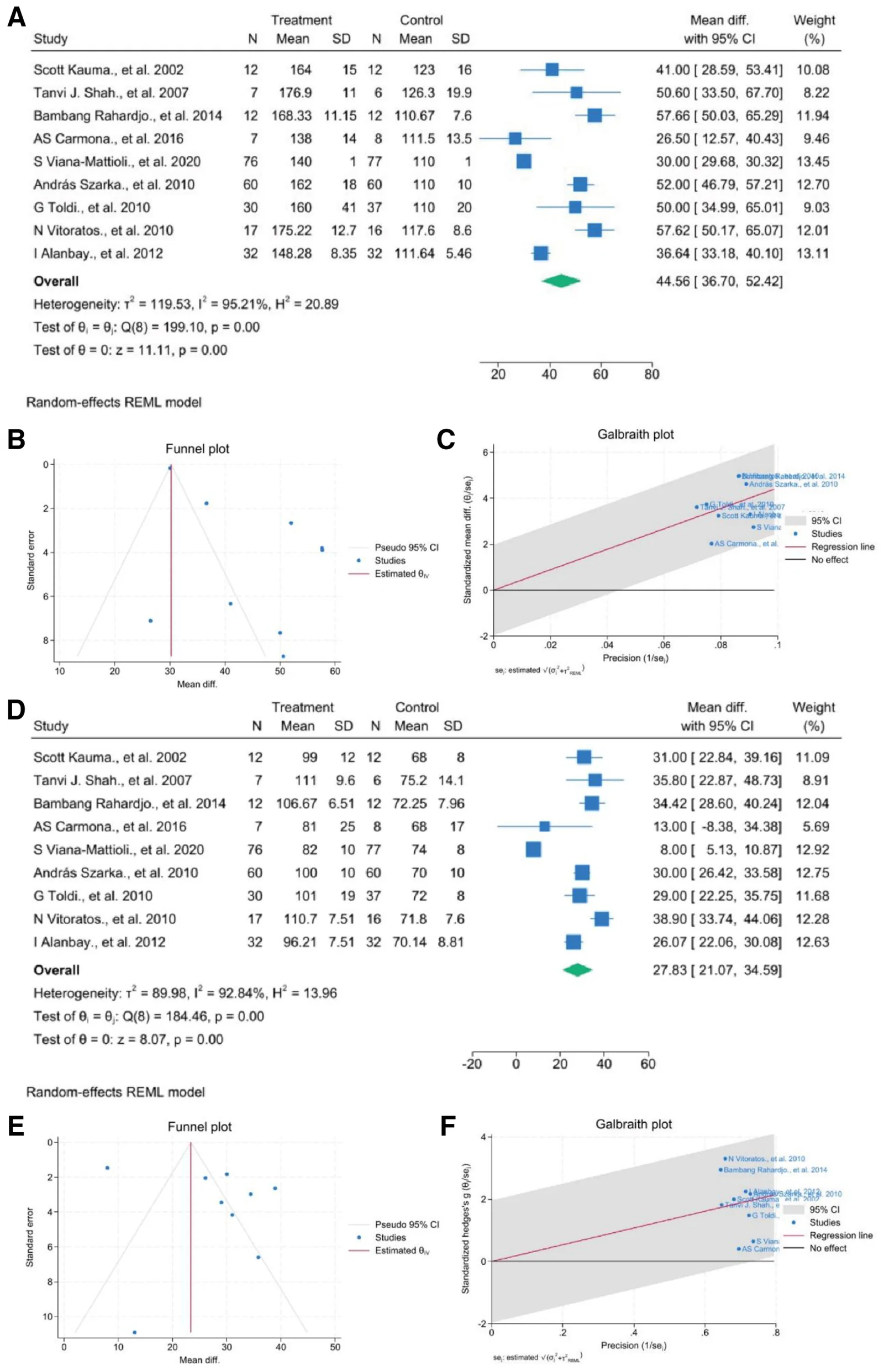
(A) Meta-analysis of preeclampsia’s impact on systolic blood pressure. (B) The funnel plot of Systolic BP. (C) Galbraith plot showing the source of the significant heterogeneity of Systolic BP. The pooled effect estimate for the overall analysis is represented by diamonds. The random-effects REML model was used to depict the data as the mean difference with 95% CI. *I*^2^ measures between-study heterogeneity with a significant *P* < .05. Study-specific effect sizes are tested for homogeneity (θi = θj), and if *P* < .05, the chi-squared test statistic is discarded. (D) Meta-analysis of preeclampsia’s impact on diastolic blood pressure. (E) The funnel plot of diastolic BP. (F) Galbraith plot showing the source of the significant heterogeneity of Diatolic BP. The pooled effect estimate for the overall analysis is represented by diamonds. The random-effects REML model was used to depict the data as the mean difference with 95% CI. *I*^2^ measures between-study heterogeneity with a significant *P* < .05. Study-specific effect sizes are tested for homogeneity (θi = θj), and if *P* < .05, the chi-squared test statistic is discarded. CI = confidence interval, REML = restricted maximum likelihood, SD = standard deviation.

In 9 trials with 9 outcomes, the impact of preeclampsia on systolic blood pressure was evaluated. These studies demonstrate a significant change in Systolic BP levels between preeclampsia and healthy women (weighted mean difference [WMD]: 44.56, 95% confidence interval [CI]: 36.70–52.42; *P* = .00; Figure 2A). Significant discrepancies across the studies resulted in notable between-study heterogeneity, as indicated by an *I*^2^ value of 95.21% (*P* < .01) (Fig. 2A). The funnel plot showed clear visual evidence of publication bias (Fig. 2B), suggesting potential small-study effects. However, Egger’s regression test, a quantitative evaluation, revealed no discernible publication bias (*P* = .5932), and Begg’s correlation test also did not demonstrate significant bias (*P* = .6022). These findings suggest that while visual inspection of the funnel plot reveals some asymmetry, significant publication bias was not confirmed by statistical tests. The Galbraith plot (Fig. 2C) illustrates that all analyzed studies fall within the 95% CI, suggesting that the estimated effects are consistent across studies despite the noted heterogeneity.

**Figure 3.**
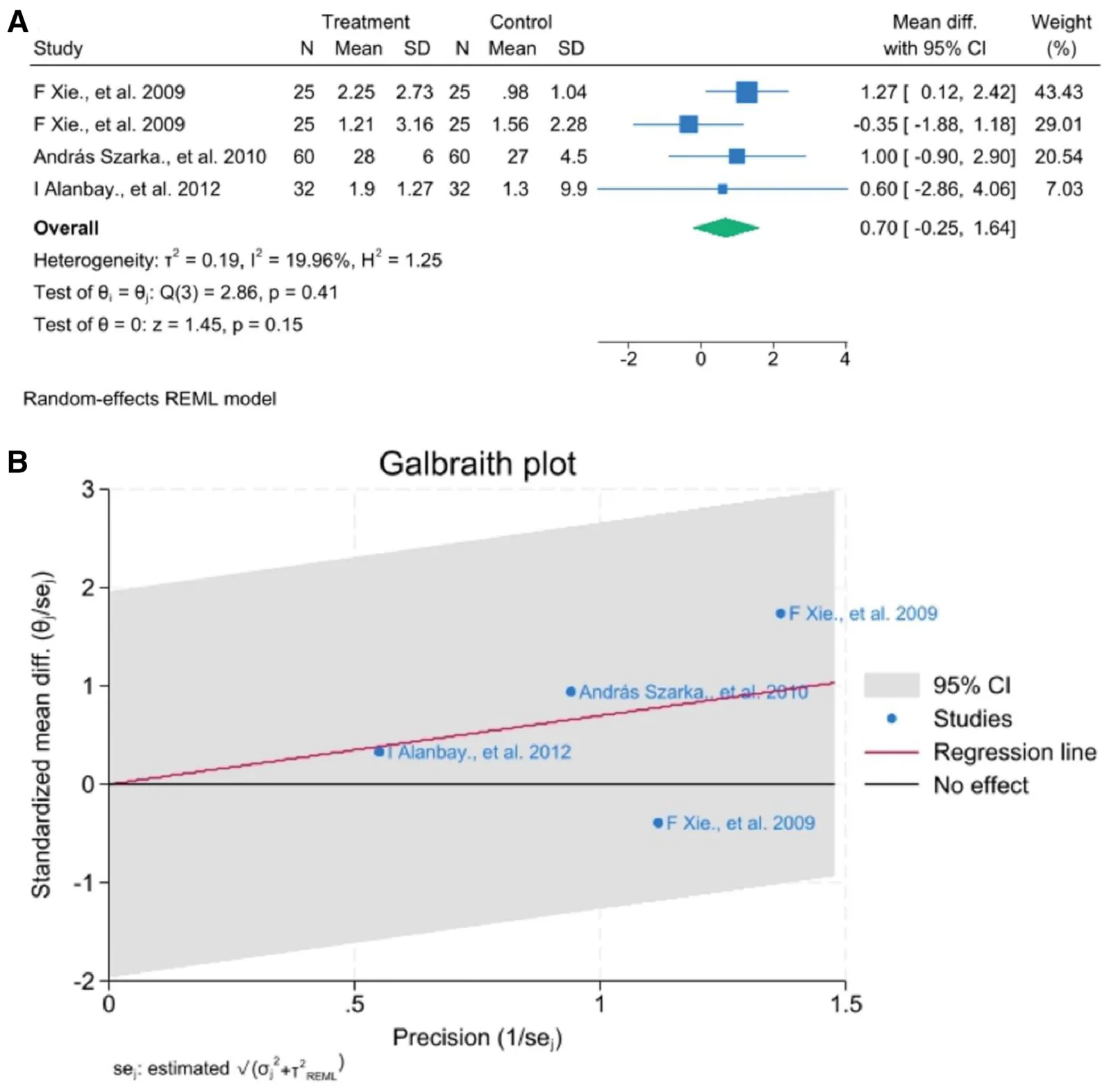
(A) Meta-analysis of the effect of preeclampsia on IL-1β. (B) Galbraith plot showing the source of the significant heterogeneity of IL-1β. The pooled effect estimate for the overall analysis is represented by diamonds. The random-effects REML model was used to depict the data as the mean difference with 95% CI. *I*^2^ measures between-study heterogeneity, with *P* < .05 considered significant. Study-specific effect sizes are tested for homogeneity (θi = θj), and if *P* < .05, the chi-squared test statistic is discarded. CI = confidence interval, SD = standard deviation.

The effect of Preeclampsia on Diastolic BP was assessed in 9 studies, encompassing 9 outcomes. These studies show a notable change in Diastolic BP levels between preeclampsia and healthy women (WMD: 27.83, 95% CI: 21.07 to 34.59; *P* = .00; Figure 3A). Significant between-study heterogeneity resulted from a noteworthy discrepancy among these investigations, with an I^2^ value of 92.84% (*P* < .01) (Fig. 2D). The funnel plot indicated that there was publication bias (Fig. 2E); however, Egger’s regression test (*P* = .9384) and Begg’s correlation test (*P* = .9170) did not support it. The Galbraith plot (Fig. 2F) demonstrates that all the studies analyzed are contained within the 95% CI, showing consistency in the estimated effects across studies, despite significant heterogeneity.

### 3.4. The impact of preeclampsia on inflammatory markers

The effect of Preeclampsia on interleukin-1 beta (IL-1β) was assessed in 3 studies, including 4 outcomes. Meta-analysis shows no difference between pregnant women with preeclampsia and normal pregnant women (95% CI [−0.25, 1.64], *P* = .15; Figure 3A). Despite some variability among the studies, there is minimal between-study heterogeneity, with an I^2^ value of 19.96%. Egger’s regression test revealed no evidence of publication bias (*P* = .8024), and Begg’s correlation test (*P* = .7341). The Galbraith plot (Fig. 3B) demonstrates that all the studies analyzed are contained within the 95% CI, showing consistency in the estimated effects across studies.

**Figure 4.**
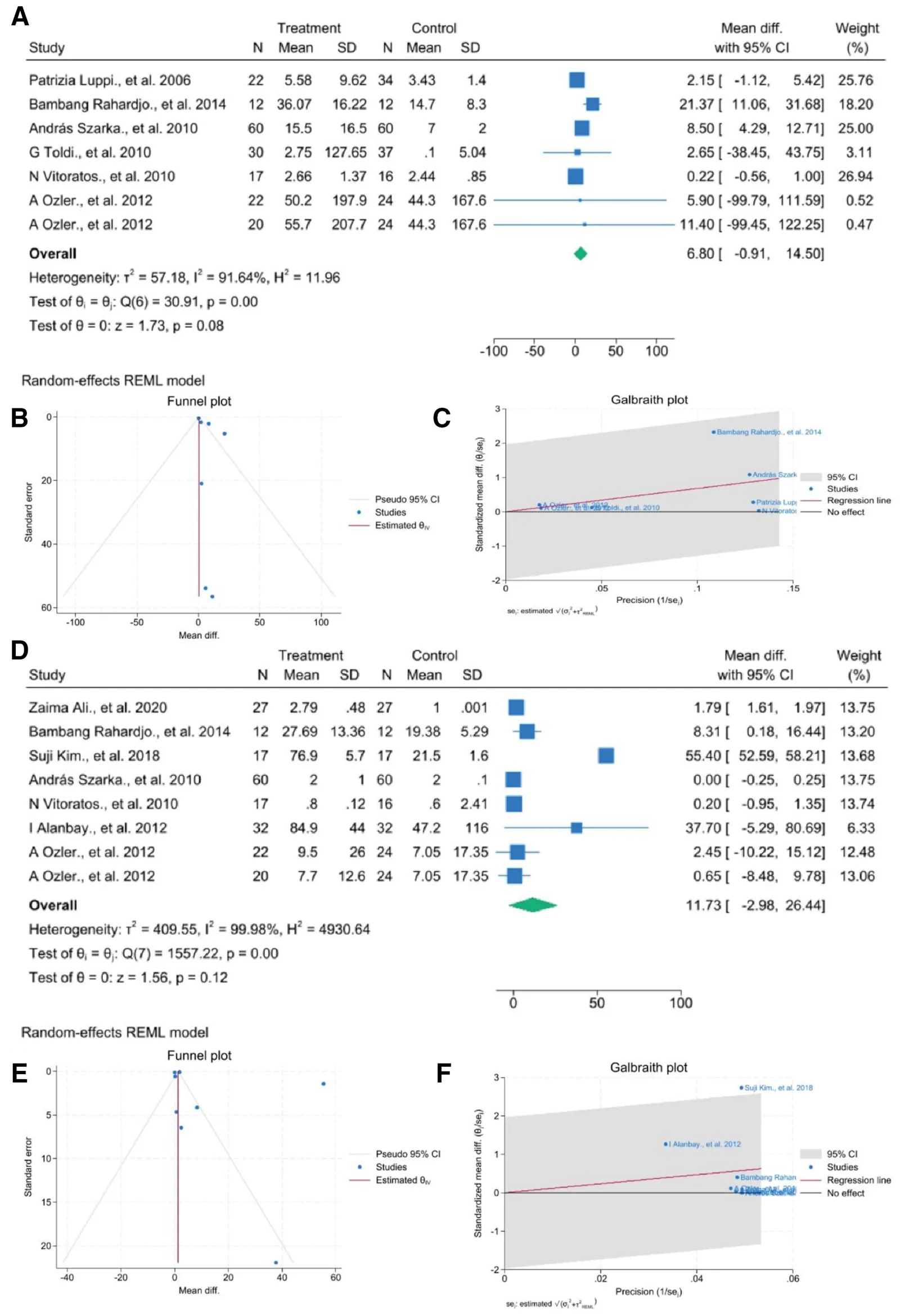
(A) Meta-analysis of preeclampsia’s impact on IL-6. (B) The IL-6 funnel plot. (C) A Galbraith plot illustrating the cause of the notable variation in IL-6. The pooled effect estimate for the overall analysis is represented by diamonds. The random-effects REML model was used to depict the data as the mean difference with 95% CI. *I*^2^ measures between-study heterogeneity with a significant *P* < .05. Study-specific effect sizes are tested for homogeneity (θi = θj), and if *P* < .05, the chi-squared test statistic is discarded. (D) Meta-analysis of the effect of preeclampsia on TNF-*α*. (E) The funnel plot of TNF-*α*. (F) Galbraith plot showing the source of the significant heterogeneity of TNF-α. The pooled effect estimate for the overall analysis is represented by diamonds. The random-effects REML model was used to depict the data as the mean difference with 95% CI. *I*^2^ measures between-study heterogeneity, with *P* < .05 considered significant. Study-specific effect sizes are tested for homogeneity (θi = θj), and if *P* < .05, the chi-squared test statistic is discarded. CI = confidence interval, SD = standard deviation.

**Figure 5.**
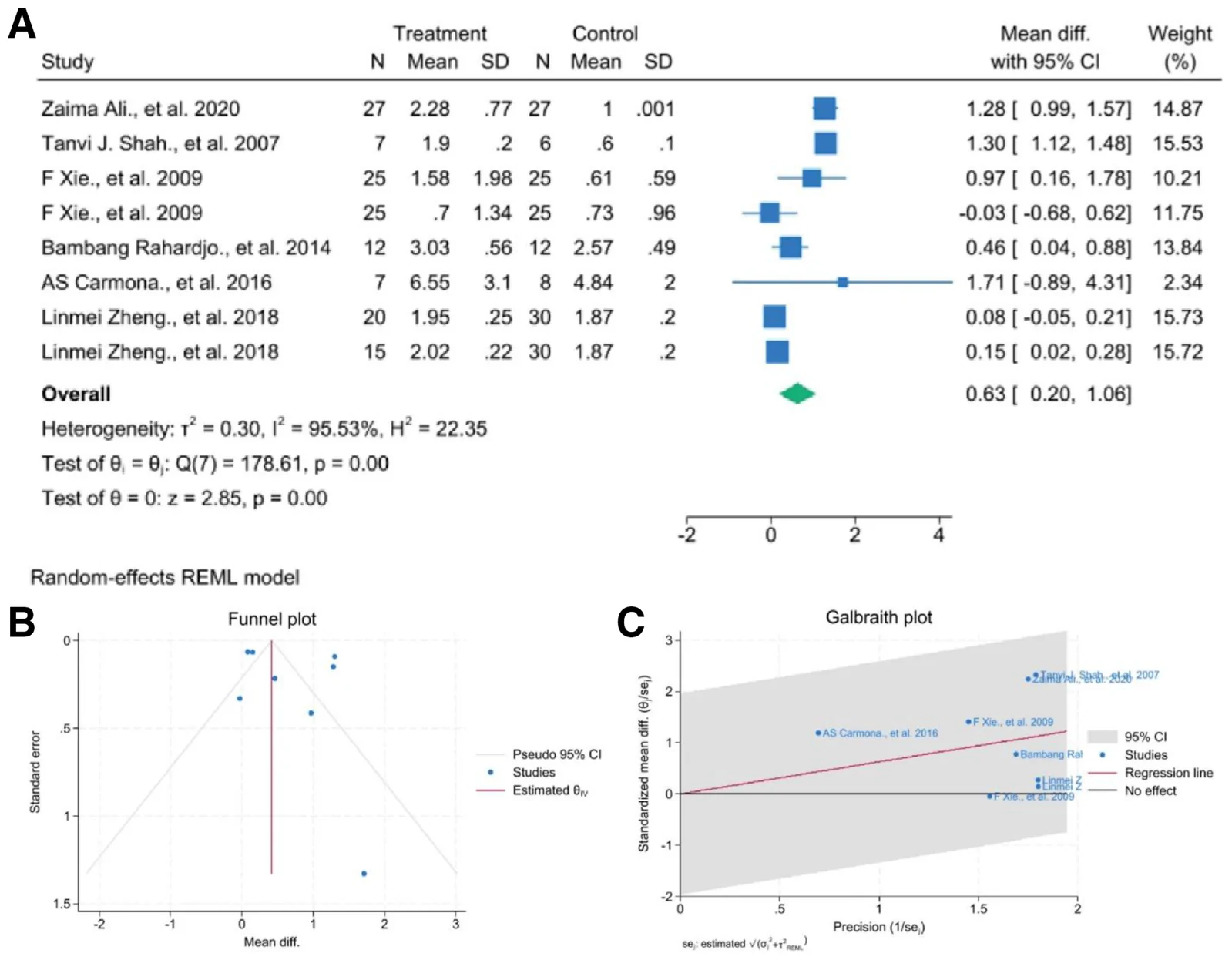
(A) Meta-analysis of the effect of preeclampsia on NFκB. (B) The funnel plot of NFκB. (C) Galbraith plot showing the source of the significant heterogeneity of NFκB. The pooled effect estimate for the overall analysis is represented by diamonds. The random-effects REML model was used to depict the data as the mean difference with 95% CI. *I*^2^ measures between-study heterogeneity, with *P* < .05 considered significant. Study-specific effect sizes are tested for homogeneity (θi = θj), and if *P* < .05, the chi-squared test statistic is discarded. CI = confidence interval, SD = standard deviation.

Six studies assessed the impact of preeclampsia on IL-6 levels (7 outcomes). However, these studies did not reveal any significant difference (WMD: 6.8, 95% CI: -0.91, 14.50, *P* = .08; Figure 4A). There was substantial between-study heterogeneity (*I*^2^ = 91.64%, *P* = .00, Figure 5A). A funnel plot was used to identify publication bias (Fig. 4B), but it did not appear in Begg’s correlation test (*P* = .7639) or Egger’s regression test (*P* = .6458). Also, no source of heterogeneity was found by our Galbraith plot (Fig. 4C).

The effect of preeclampsia on TNF-α level was evaluated in 7 studies (8 outcomes). These studies showed a significant difference (WMD: 11.73, 95% CI: −2.98, 26.44, *P* < .01; Figure 4D) between the preeclampsia pregnant women and healthy pregnant women. There was substantial between-study heterogeneity (*I*^2^ = 99.98%, *P* = .00, Figure 4D). Publication bias was found by the funnel plot (Fig. 4E), but was not found in Egger’s regression test (*P* = .4948) and Begg’s correlation test (*P* = .9015). Our Galbraith plot found 1 source of heterogeneity. However, the total heterogeneity did not significantly decrease after eliminating this study (*I*^2^ = 93.45%) (Fig. 4F), suggesting that various researchers reported contradictory findings (Figure S1, Supplemental Digital Content 2).

The effect of preeclampsia on NF-κB level was evaluated in 6 studies (8 outcomes). These studies showed a significant difference (WMD: 0.63, 95% CI: 0.20, 1.06, *P* < .01; Figure 5A) between the preeclampsia pregnant women and healthy pregnant women. There was substantial between-study heterogeneity (*I*^2^ = 95.53%, *P* = .00, Figure 5A). Publication bias was not found by the funnel plot (Fig. 5B), also in Egger’s regression test (*P* = .5046), and Begg’s correlation test (*P* = .5362). In Figure 5C, the source of heterogeneity was not identified by our Galbraith plot.

## 4. Discussion

By combining information from 15 research, this meta-analysis thoroughly investigates the function of NF-κB in the pathophysiology of preeclampsia, revealing significant insights into how NF-κB and associated inflammatory markers influence the progression of the disease. The analysis indicates significant elevations in both systolic and diastolic blood pressures, showing the hypertensive symptoms integral to preeclampsia. While hypertension is not directly an inflammatory marker, its consistent association with elevated NF-κB activity in this analysis shows the interconnected roles of inflammation and vascular regulation in the pathology of the disease. The elevation of NF-κB as well as TNF-α observed in our analysis correlates strongly with the inflammatory environment typical of preeclampsia.^[23]^ While IL-1β and IL-6 did not show significant changes, the trend towards increased IL-6 levels, although not statistically significant, suggests its potential involvement. In addition, SIRT1 is another regulator of inflammatory responses. Despite the limited data on SIRT1, preventing a comprehensive analysis of its roles, it is important to discuss the theoretical implications of SIRT1 in inflammation related to preeclampsia. SIRT1 is known for its role in deacetylating various substrates including NF-κB, thereby potentially moderating the inflammatory response.^[24]^ Its interaction with NF-κB suggests a complex regulatory mechanism that could influence the severity and outcomes of preeclampsia.

Activating NF-κB is crucial for mediating the inflammatory responses characteristic of preeclampsia, coordinating a complex interplay of molecular pathways that exacerbate the condition’s pathology.^[25]^ This transcription factor regulates many genes involved in the immune response, including cytokines such as TNF-α, IL-6, and IL-1β.^[26,27]^ Our meta-analysis reveals significant elevations not only in NF-κB but also in TNF-α levels in preeclamptic patients, which indicates inflammatory states. The elevated levels of these cytokines contribute to a pro-inflammatory state that exacerbates endothelial dysfunction, which is also a key pathological feature of preeclampsia; elevated TNF-α, facilitated by NF-κB activation, further disrupts endothelial cell function, leading to increased vascular permeability and hypertension.^[15]^ Additionally, the involvement of SIRT1 in the inflammatory pathways offers an intriguing counterbalance to NF-κB activity.^[28]^ SIRT1, known for its anti-inflammatory properties, acts through the deacetylation of several key transcription factors, including NF-κB, which could modulate the inflammatory cascade pivotal in preeclampsia.^[28–30]^ Despite the lack of enough data to analyze SIRT1, it is still an important factor in studying preeclampsia. SIRT1’s potential to repress NF-κB activity suggests that enhancing SIRT1 activity could be a novel approach to mitigate the inflammatory responses. This modulation is particularly critical given that SIRT1 also influences other metabolic and stress-related pathways, which might be dysregulated in preeclampsia.

Further, NF-κB’s interaction with various signalling pathways amplifies its effects in preeclampsia. For instance, the interplay between NF-κB and the angiotensin II type 1 receptor autoantibody (AT1-AA) suggests a mechanism that potentiates inflammatory and hypertensive responses. AT1-AA has been implicated in the modulation of NF-κB activity, leading to increased sensitivity to angiotensin II, which is known to contribute to hypertension: a primary symptom of preeclampsia.^[31]^ In addition, oxidative stress also plays a crucial role in the activation of NF-κB. The oxidative environment in preeclamptic patients triggers the NF-κB pathway through the activation of IκB Kinase (IKK) complex, the induction of the IKK complex triggers the degradation of inhibitor of kappa B, leading to the nuclear translocation of NF-κB and further promoting its transcriptional activity on inflammatory genes.^[14,32]^ This oxidative-induced NF-κB activation suggests that antioxidant therapies might attenuate the inflammatory response by modulating NF-κB activity. The pivotal role of NF-κB in driving the inflammatory process in preeclampsia may open new avenues for therapeutic intervention. Targeting NF-κB and its upstream activators could potentially mitigate the inflammatory and hypertensive aspects of preeclampsia. For example, the use of specific inhibitors like IKK inhibitors, which prevent the activation of NF-κB, could be explored.^[33]^ Additionally, therapies aimed at reducing oxidative stress or blocking AT1-AA could indirectly decrease NF-κB activation, offering a multifaceted approach to treatment.^[34]^ However, translating these findings into clinical practice involves several challenges. The NF-κB’s role in cell signaling and immunity means that targeting this pathway should be approached with caution to avoid unintended immunosuppressive effects, particularly in the pregnancy women. Moreover, the timing of therapeutic intervention is crucial; early in pregnancy, interventions might prevent the onset of severe symptoms, but later interventions could be less effective or even detrimental.^[35]^

The heterogeneity observed in this study outcomes indicate the complexity of NF-κB’s role in preeclampsia. This could be attributed to several factors, including the wide age range of the pregnant women involved, and the severity of preeclampsia, as studies included both mild and severe cases. Age is a critical factor; younger and older mothers may exhibit different biological responses to pregnancy, potentially affecting the expression levels of inflammatory markers like NF-κB. In most cases, older women often have a higher risk of pregnancy complications, which might amplify inflammatory responses.^[36]^ Similarly, the severity of preeclampsia impacts NF-κB activity; severe cases are likely to exhibit more pronounced inflammatory responses, contributing to greater variability in the data. Additionally, genetic diversity and underlying health conditions, such as preexisting hypertension or diabetes, could also influence NF-κB activity. These conditions can alter the baseline inflammatory status of the individuals, leading to variability in how NF-κB is expressed during preeclampsia. Lifestyle factors, such as diet and smoking, could also modulate inflammatory responses and thus NF-κB activity.^[37]^ Future research should prioritize longitudinal studies that track NF-κB activity from early pregnancy. This would help in understanding the temporal dynamics of NF-κB, illuminating how its activity changes throughout pregnancy and in response to the progression of preeclampsia, which is important for earlier and more effective management of preeclampsia. Given the limited number of studies available for this meta-analysis, this field requires more extensive research to fully understand the dynamics of NF-κB activity in preeclampsia. Moreover, more research is needed to elucidate the specific contributions of SIRT1 and to integrate these insights into a broader understanding of the molecular mechanisms driving preeclampsia. In the future, research should focus more on increasing the frequency of observations, incorporating comprehensive genomic and metabolomic approaches. By analyzing large clinical samples using transcriptomics and metabolomics, researchers can identify biomarkers and pathways that correlate with changes in NF-κB activity. Furthermore, expanding the scope of these studies to include diverse populations and varying degrees of preeclampsia severity wilThe substantial heterogeneity observed in several outcomes may be explained by both methodological and biological differences across the included studies. First, differences in detection methods may have contributed to variability in the measured levels of inflammatory markers. For example, NF-κB activity and cytokine concentrations may be evaluated using different laboratory techniques, including enzyme-linked immunosorbent assay, immunohistochemistry, Western blotting, polymerase chain reaction-based methods, or other quantitative platforms. These methods differ in sensitivity, specificity, normalization procedures, and the biological level measured, such as protein expression, gene expression, or transcriptional activation.

Second, the type of biological sample used in each study may also influence the results. Some studies measured inflammatory markers in maternal serum or plasma, whereas others focused on placental tissue, peripheral blood mononuclear cells, or other pregnancy-related specimens. Circulating samples may better reflect systemic maternal inflammation, whereas placental samples may reflect local inflammatory activation at the maternal–fetal interface. Therefore, differences in sample source may partly explain the inconsistent magnitude of changes in NF-κB, TNF-α, IL-1β, and IL-6 across studies.

Third, regional and population differences should be considered. The included studies were conducted in different geographical regions and may have involved populations with different ethnic backgrounds, nutritional status, environmental exposures, access to antenatal care, and clinical management strategies. In addition, variations in diagnostic criteria, gestational age at sampling, early- versus late-onset preeclampsia, and mild versus severe disease may further contribute to heterogeneity. Maternal age, body mass index, parity, preexisting hypertension, diabetes, and lifestyle factors may also influence baseline inflammatory status and NF-κB pathway activation. These factors should be carefully considered when interpreting the pooled estimates, and future studies should report detection methods, sample sources, disease severity, and population characteristics in greater detail to facilitate subgroup analyses.l help in understanding the differential expression and impact of NF-κB.

## 5. Conclusion

In conclusion, our meta-analysis emphasizes the crucial role of NF-κB and associated inflammatory markers in the pathology of preeclampsia. Our findings show the significant upregulation of NF-κB and TNF-α in this condition, pointing to their central roles in exacerbating the inflammatory processes. While tremendous progress has been made in understanding this complex disease, additional research is required to translate these results into useful treatment solutions. This will ultimately improve outcomes for both mothers and their infants, addressing a major public health challenge.

## Author contributions

**Conceptualization:** Hui Zhang, Yanhua Nong, Meiqi Huang, Riyuan Wei, Lihan Feng.

**Data curation:** Hui Zhang, Yanhua Nong, Meiqi Huang, Riyuan Wei, Lihan Feng.

**Formal analysis:** Hui Zhang, Yanhua Nong, Meiqi Huang, Riyuan Wei, Lihan Feng.

**Funding acquisition:** Hui Zhang.

**Investigation:** Hui Zhang.

**Writing – original draft:** Hui Zhang.

**Writing – review & editing:** Hui Zhang.




