# Supplementary material for: The NF-κB pathway in inflammatory responses in preeclampsia: A systematic review and meta-analysis
Source: Medicine (Baltimore). 2026 Sep 18;105(38):e50552. doi: 10.1097/MD.0000000000050552 (PMC13593243; doi:10.1097/MD.0000000000050552)
Supplement: Supplementary file 1 [file medi-105-e50552-s001.docx]

Table S1 The characteristics of the included studies

| Reference | n | Maternal age (years) | Gestational age (weeks) | Parity | BMI | Gender | Health condition | Study design | Comparator | Duration |
| --- | --- | --- | --- | --- | --- | --- | --- | --- | --- | --- |
| [1] | 54 | 23-30 | 28-36 | 1-2 | 23.8-30.3 | F | preeclampsia | case-control study | normotensive pregnant women | third trimester (28-40 weeks) |
| [2] | 24 | 24-25 | 35.7-38.1 | 1 | / | F | severe preeclampsia | cross-sectional study | Normal pregnant women | less than 32 weeks |
| [3] | 13 | 22-25 | 33.3-38.8 | 1 | 30.8-31.2 | F | 4 had severe preeclampsia, 6 delivered preterm, and 3 had IUGR. | case series or a cohort study | Normal pregnant women | / |
| [4] | 56 | 15-42 | / | / | / | F | preeclampsia | cross-sectional study | Normal pregnant women | 33.8±3.6 weeks |
| [5] | 50 | 24-43 | / | / | / | F | early-onset pre-eclampsia | case–control study | women with normal pregnancy | <34 weeks |
| [5] | 50 | 23-41 | / | / | / | F | late-onset pre-eclampsia | case–control study | women with normal pregnancy | ≥34 weeks |
| [6] | 24 | 25.08-29 | 36.5-38.08 | / | 27.54-33.26 | F | [severe preeclampsia (late onset)](https://www.sciencedirect.com/topics/pharmacology-toxicology-and-pharmaceutical-science/preeclampsia) | experimental study | normotensive primigravidae | <34 weeks |
| [7] | 15 | 17-29 | / | 1-2 | 26.2-28.6 | F | preeclampsia | cross-sectional, prospective, nonrandomized, and descriptive study | women with normal pregnancy | / |
| [8] | 34 | / | / | / | / | F | preeclampsia | prospective cohort study | women with normal pregnancy | / |
| [9] | 50 | / | / | / | / | F | mild preeclampsia | case–control study | women with normal pregnancy | after 20 weeks |
| [9] | 45 | / | / | / | / | F | severe preeclampsia | case–control study | women with normal pregnancy | after 20 weeks |
| [10] | 34 | 26-28 | 34-36 | / | 27-34 | F | preeclampsia | case–control study | women with normal pregnancy | / |
| [11] | 120 | 26-32 | 36-37 | / | 25.8-29.9 | F | preeclampsia | case–control study | Healthy pregnant women | <34 weeks |
| [12] | 67 | 17-45 | 24-40 | / | / | F | preeclampsia | case–control study | Healthy pregnant women | / |
| [13] | 33 | 31.14-33.57 | / | / | 25-28.96 | F | preeclampsia | case–control study | Healthy pregnant women | / |
| [14] | 64 | 27.2-27.31 | / | 1.42-1.49 | / | F | Mild preeclampsia | case–control study | Healthy pregnant women | / |
| [15] | 46 | 30.3-32.3 | 34.3-34.7 | / | 28.2-31.1 | F | Mild preeclampsia | case–control study | Healthy pregnant women | / |
| [15] | 44 | 29.2-32.3 | 32.7-34.7 | / | 28.2-28.3 | F | severe preeclampsia | case–control study | Healthy pregnant women | / |

Reference:

1.	Ali, Z., et al., *Elevated Tumor Necrosis Factor (TNF)-alpha mRNA Expression Correlates with Nuclear Factor Kappa B Expression in Peripheral Blood Mononuclear Cells in Preeclampsia.* Jcpsp, Journal of the College of Physicians & Surgeons - Pakistan, 2020. **30**(2): p. 158-162.

2.	Kauma, S., et al., *Increased endothelial monocyte chemoattractant protein-1 and interleukin-8 in preeclampsia.* Obstetrics and Gynecology, 2002. **100**(4): p. 706-714.

3.	Shah, T.J. and S.W. Walsh, *Activation of NF-κB and expression of COX-2 is associated with neutrophil infiltration in systemic vascular tissue of women with preeclampsia.* Journal of the Society for Gynecologic Investigation, 2005. **12**(2): p. 196A-196A.

4.	Luppi, P., et al., *Preeclampsia activates circulating immune cells with engagement of the NF-<i>κ</i>B pathway.* American Journal of Reproductive Immunology, 2006. **56**(2): p. 135-144.

5.	Xie, F., et al., *Toll-like receptors 2 and 4 and the cryopyrin inflammasome in normal pregnancy and pre-eclampsia.* Bjog-an International Journal of Obstetrics and Gynaecology, 2010. **117**(1): p. 99-108.

6.	Rahardjo, B., et al., *Curcumin decreased level of proinflammatory cytokines in monocyte cultures exposed to preeclamptic plasma by affecting the transcription factors NF-κB and PPAR-γ.* Biomarkers and Genomic Medicine, 2014. **6**(3): p. 105-115.

7.	Silva Carmona, A. and H. Mendieta Zerón, *NF-κΒ and SOD expression in preeclamptic placentas.* Turk J Med Sci, 2016. **46**(3): p. 783-8.

8.	Kim, S., et al., *NF-κB-responsive miRNA-31-5p elicits endothelial dysfunction associated with preeclampsia via down-regulation of endothelial nitric-oxide synthase.* Journal of Biological Chemistry, 2018. **293**(49): p. 18989-19000.

9.	Zheng, L., et al., *Placental expression of AChE, alpha7nAChR and NF-kappaB in patients with preeclampsia.* Ginekologia Polska, 2018. **89**(5): p. 249-255.

10.	Viana-Mattioli, S., et al., *Analysis of SIRT1 Expression in Plasma and in an <i>In Vitro</i> Model of Preeclampsia.* Oxidative Medicine and Cellular Longevity, 2020. **2020**.

11.	Szarka, A., et al., *Circulating cytokines, chemokines and adhesion molecules in normal pregnancy and preeclampsia determined by multiplex suspension array.* BMC immunology, 2010. **11**: p. 1-9.

12.	Toldi, G., et al., *Hepcidin concentrations and iron homeostasis in preeclampsia.* Clinical chemistry and laboratory medicine, 2010. **48**(10): p. 1423-1426.

13.	Vitoratos, N., et al., *Maternal serum levels of TNF‐alpha and IL‐6 long after delivery in preeclamptic and normotensive pregnant women.* Mediators of inflammation, 2010. **2010**(1): p. 908649.

14.	Alanbay, I., et al., *Chitotriosidase, interleukin-1 beta and tumor necrosis factor alpha levels in mild preeclampsia.* Archives of gynecology and obstetrics, 2012. **285**: p. 1505-1511.

15.	Ozler, A., et al., *Serum levels of neopterin, tumor necrosis factor-alpha and Interleukin-6 in preeclampsia: relationship with disease severity.* Eur Rev Med Pharmacol Sci, 2012. **16**(12): p. 1707-12.
